# Brain magnetic resonance imaging outperforms clinical severity ratings in the prediction of treatment outcomes in major depressive disorder

**DOI:** 10.1192/j.eurpsy.2024.219

**Published:** 2024-08-27

**Authors:** F. Long, Y. Chen, Q. Zhang, Y. Wang, Y. Wang, Q. Li, Y. Zhao, F. Li

**Affiliations:** Department of Radiology and Huaxi MR Research Center (HMRRC), Functional and Molecular Imaging Key Laboratory of Sichuan Province, West China Hospital, Sichuan University, Chengdu 610041, Sichuan Province, China

## Abstract

**Introduction:**

Major depressive disorder (MDD) is a prevalent and disabling condition. Approximately 30-50% of patients do not respond to first-line medication or psychotherapy. Therefore, several studies have investigated the predictive potential of pretreatment severity rating or neuroimaging features to guide clinical approaches that can speed optimal treatment selection.

**Objectives:**

To evaluate the performance of 1) severity ratings (scores of Hamilton Depression/Anxiety Scale, illness duration, and sleep quality, etc.) and demographic characteristic and 2) brain magnetic resonance imaging (MRI) features in predicting treatment outcomes for MDD. Second, to assess performance variations among varied modalities and interventions in MRI studies.

**Methods:**

We searched studies in PubMed, Embase, Web of Science, and Science Direct databases before March 22, 2023. We extracted a confusion matrix for prediction in each study. Separate meta-analyses were performed for clinical and MRI studies. The logarithm of diagnostic odds ratio [log(DOR)], sensitivity, and specificity were conducted using Reitsma’s random effect model. The area under curve (AUC) of summary receiver operating characteristic (SROC) curve was calculated.

Subgroup analyses were conducted in MRI studies based on modalities: resting-state functional MRI (rsfMRI), task-based fMRI (tbfMRI), and structural MRI (sMRI), and interventions: antidepressant (including selective serotonin reuptake inhibitors [SSRI]) and electroconvulsive therapy (ECT). Meta-regression was conducted 1) between clinical and MRI studies and 2) among modality or intervention subgroups in MRI studies.

**Results:**

We included ten studies used clinical features covering 6494 patients, yielded a log(DOR) of 1.42, AUC of 0.71, sensitivity of 0.61, and specificity of 0.74. In terms of MRI, 44 studies with 2623 patients were included, revealing an overall log(DOR) of 2.53. The AUC, sensitivity, and specificity were 0.89, 0.78, and 0.75.

Studies using MRI features had a higher sensitivity (0.89 vs. 0.61) in predicting treatment outcomes than clinical features (*P* < 0.001). RsfMRI had higher specificity (0.79 vs. 0.69) than tbfMRI subgroup (*P* = 0.01). No significant differences were found between sMRI and other modalities, nor between antidepressants (SSRIs and others) and ECT. Antidepressant studies primarily identified predictive imaging features in limbic and default mode networks, while ECT mainly focused on limbic network.

**Conclusions:**

Our findings suggest a robust promise for pretreatment brain MRI features in predicting treatment outcomes in MDD, offering higher accuracy than clinical studies. While tasks in tbfMRI studies differed, those studies overall had less predictive utility than rsfMRI data. For MRI studies, overlapping but distinct network level measures predicted outcomes for antidepressants and ECT.

**Disclosure of Interest:**

None Declared

